# Protocol for detection and monitoring of post-stroke cognitive impairment through AI-powered speech analysis: a mixed methods pilot study

**DOI:** 10.3389/fnagi.2025.1581891

**Published:** 2025-05-01

**Authors:** Ravi Shankar, Effie Chew, Anjali Bundele, Amartya Mukhopadhyay

**Affiliations:** ^1^Medical Affairs – Research Innovation & Enterprise, Alexandra Hospital, National University Health System, Singapore, Singapore; ^2^Division of Rehabilitation Medicine, Department of Medicine, National University Hospital, Singapore, Singapore; ^3^Department of Medicine, Yong Loo Lin School of Medicine, National University of Singapore, Singapore, Singapore; ^4^Division of Rehabilitation Medicine, Department of Medicine, Alexandra Hospital, Singapore, Singapore; ^5^Yong Loo Lin School of Medicine, National University of Singapore, Singapore, Singapore; ^6^Division of Respiratory & Critical Care Medicine, Department of Medicine, National University Health System, Singapore, Singapore

**Keywords:** post-stroke cognitive impairment, natural language processing, speech analysis, digital biomarkers, cognitive screening, machine learning, mixed methods

## Abstract

**Introduction:**

Post-stroke cognitive impairment (PSCI) affects up to 75% of stroke survivors but remains challenging to detect with traditional neuropsychological assessments. Recent advances in artificial intelligence and natural language processing have opened new avenues for cognitive screening through speech analysis, yet their application to PSCI remains largely unexplored. This study aims to characterize speech markers of PSCI in the first-year post-stroke and evaluate their utility for predicting cognitive outcomes in a Singapore cohort.

**Methods:**

This prospective mixed-methods study will recruit 30 stroke survivors from the Alexandra Hospital and National University Hospital in Singapore. Participants will be assessed at four timepoints: baseline (within 6 weeks of stroke onset), 3-, 6-, and 12-months post-stroke. At each visit, participants will complete the Montreal Cognitive Assessment (MoCA) and a standardized speech protocol comprising picture description and semi-structured conversation tasks. Speech recordings will be automatically transcribed using automated speech recognition (ASR) systems based on pretrained acoustic models, and comprehensive linguistic and acoustic features will be extracted. Machine learning models will be developed to predict MoCA-defined cognitive impairment. Statistical analysis will include correlation analysis between speech features and MoCA scores, as well as machine learning classification and regression models to predict cognitive impairment. Linear mixed-effects models will characterize trajectories of MoCA scores and speech features over time. Qualitative analysis will follow an inductive thematic approach to explore acceptability and usability of speech-based screening.

**Discussion:**

This study represents a critical step toward developing speech-based digital biomarkers for PSCI detection that are sensitive, culturally appropriate, and clinically feasible. If validated, this approach could transform current models of PSCI care by enabling remote, frequent, and naturalistic monitoring of cognitive health, potentially improving outcomes through earlier intervention.

## Introduction

Post-stroke cognitive impairment (PSCI) affects up to 75% of stroke survivors (Lee et al., 2023; Alhashimi et al., 2024) but remains challenging to detect and monitor using traditional neuropsychological assessments (Tang et al., 2018). PSCI can span multiple cognitive domains and progress to dementia, leading to poorer functional outcomes, reduced quality of life, and increased caregiver burden (Sun et al., 2014; Pendlebury and Rothwell, 2009). Early identification is critical for timely intervention and support (Kalaria et al., 2016), but current screening tools often lack sensitivity to subtle deficits (Zietemann et al., 2018).

Recent advances in artificial intelligence (AI), natural language processing (NLP), and automatic speech recognition (ASR) have opened new avenues for detecting cognitive decline through speech analysis (Asgari et al., 2017). Studies in Alzheimer’s disease and mild cognitive impairment have found that linguistic features extracted from spontaneous speech can predict cognitive status with high accuracy (Fraser et al., 2016; König et al., 2015; Beltrami et al., 2018). These “digital biomarkers” offer several advantages over traditional assessments, including greater objectivity, ecological validity, and suitability for remote, frequent monitoring (de la Fuente et al., 2020; Robin et al., 2020). However, their application to PSCI remains largely unexplored.

Developing speech-based screening tools is particularly pertinent in Singapore, which faces a rapidly aging population and increasing stroke burden (Venketasubramanian et al., 2017; Teh et al., 2018). Current approaches relying on brief cognitive tests or full neuropsychological batteries are limited by suboptimal sensitivity, need for specialized training, and infrequent administration (Zhao et al., 2020). Automated speech analysis could provide a scalable, cost-effective solution for detecting PSCI in routine clinical care and research (Mirheidari et al., 2024; Simon et al., 2024; Martínez-Nicolás et al., 2021; Akkad et al., 2023). Adapting these tools for Singapore’s multilingual context could also help address linguistic and cultural gaps in cognitive assessment.

This pilot study aims to characterize speech markers of PSCI in the first-year post-stroke and evaluate their utility for predicting cognitive outcomes in a Singapore cohort. Unlike Alzheimer’s disease (AD), which typically follows a predictable progression pattern primarily affecting memory systems initially, PSCI presents with heterogeneous cognitive profiles influenced by stroke location, size, and type. We anticipate observing distinct linguistic-cognitive patterns in PSCI patients compared to those with AD. Specifically, we expect to detect: (1) reduced information content and coherence reflecting executive dysfunction commonly seen after stroke; (2) impaired word retrieval and semantic processing manifesting as word-finding difficulties and circumlocutions; (3) syntactic simplification correlating with working memory deficits; and (4) prosodic alterations reflecting frontal-subcortical pathway disruptions.

Recent work by Mirheidari et al. (2024) has demonstrated the feasibility of detecting cognitive impairment in stroke survivors through speech analysis, showing that both acoustic features (particularly emotion-based prosodic features) and linguistic features (especially those capturing contextual information) can effectively predict cognitive status. These speech markers are clinically significant as they may emerge before traditional screening tools detect impairment, particularly in highly educated individuals with cognitive reserve. By capturing these subtle linguistic changes through automated analysis, our approach could facilitate earlier detection of cognitive decline, enabling timely interventions such as cognitive rehabilitation and secondary stroke prevention to mitigate progression to dementia. Furthermore, longitudinal tracking of these markers may provide more sensitive measures of intervention efficacy than conventional assessments, supporting personalized treatment approaches.

By leveraging state-of-the-art ASR, NLP and machine learning techniques, we seek to develop a proof-of-concept speech-based screening approach that is sensitive, linguistically and culturally appropriate, and feasible for longitudinal monitoring. Specific objectives are:

1. Elicit speech samples from stroke survivors using a brief, standardized protocol comprising picture description and semi-structured conversation tasks
2. Extract linguistic and acoustic features from transcribed speech that correlate with and predict MoCA scores over 12 months post-stroke
3. Explore the acceptability and usability of speech elicitation tasks through qualitative interviews with study participants

Based on these objectives, our primary hypothesis is that speech features extracted from standardized speech tasks will correlate significantly with cognitive status as measured by MoCA scores in stroke survivors. Our secondary hypotheses are: (1) a machine learning model using speech features can predict cognitive impairment with at least 75% accuracy; (2) specific speech markers will show longitudinal changes that parallel cognitive trajectories over the 12-month follow-up period; and (3) speech-based cognitive assessment will be acceptable to stroke survivors as measured by qualitative feedback.

## Methods

### Study design and setting

This is a prospective cohort study recruiting 30 stroke survivors from the acute stroke unit and outpatient stroke clinics at the Alexandra Hospital and National University Hospital in Singapore. The study employs a longitudinal design with four assessment timepoints: baseline (within 6 weeks of stroke onset), 3 months, 6 months, and 12 months post-stroke. Stroke onset is defined as the first documented occurrence of stroke symptoms, confirmed by clinical assessment and neuroimaging.

### Visit structure and duration

The study comprises structured visits at each timepoint. Baseline visits require 90–120 min, encompassing:

- Informed consent and eligibility verification
- Clinical data collection
- MoCA assessment (15–20 min)
- Speech tasks (25–35 min total)

Follow-up visits at 3 and 6 months are shorter, lasting 60–75 min, focusing on MoCA assessment and speech tasks. The final 12-month visit includes these standard assessments plus a qualitative interview (30–45 min). Rest breaks are provided throughout all sessions as needed.

At each visit, participants complete the Montreal Cognitive Assessment (MoCA) and standardized speech tasks. Upon study completion, all participants are invited to participate in a qualitative interview exploring their experience with the assessment protocol and its feasibility for clinical implementation.

### Participants

Inclusion Criteria:

- Age greater than or equal to 55 years
- Admitted to Alexandra Hospital and National University Hospital with acute ischemic or hemorrhagic stroke confirmed on neuroimaging
- Within 6 weeks of stroke onset
- Able to follow study procedures and provide informed consent
- Able to engage in simple conversation in English

Exclusion Criteria:

- Pre-existing diagnosis of dementia, Alzheimer’s disease, or other neurodegenerative conditions
- Severe aphasia precluding speech tasks based on clinical assessment
- Active psychiatric disorders, substance abuse, or life-limiting medical conditions

Potential participants will be screened via medical records and collaborating clinicians. Eligible individuals will be approached prior to discharge or at their first outpatient visit. Interested participants will provide written informed consent. We aim to recruit a sample of 30 over 12 months.

All study procedures will be conducted in English to ensure standardization. Participants must demonstrate functional English proficiency through basic conversation and comprehension screening. Language background including primary language, years of English education, and self-rated proficiency will be documented. This standardization is essential for the validity of speech analysis while acknowledging Singapore’s linguistic diversity.

To address Singapore’s multilingual context, our approach incorporates several adaptations. First, we are using the DeepSpeech ASR engine with transfer learning techniques to fine-tune acoustic models specifically on Singaporean English (Singlish), accounting for its unique phonological features, prosodic patterns, and lexical variations. The National Speech Corpus (NSC) provides training data representing Singapore’s ethnic diversity (Chinese, Malay, Indian, and others) and varying English proficiency levels.

Second, our linguistic feature extraction pipeline incorporates Singapore-specific linguistic resources, including locally adapted word frequency databases and lexical norms. This ensures our lexical sophistication measures reflect local language usage patterns rather than Western standards. Additionally, our syntactic complexity metrics are calibrated against Singaporean English grammatical structures, which may differ from standard English.

Third, we will employ cross-cultural validation by comparing our linguistic markers against previously published norms, adjusting thresholds and interpretations as needed. Qualitative interviews will further explore cultural factors affecting speech task performance, informing future refinements.

We recognize that bilingualism and multilingualism are defining features of Singapore’s linguistic landscape, with participants likely to have varying degrees of proficiency across multiple languages. While conducting the study in English provides standardization, we acknowledge that code-switching (alternating between languages within conversation) and accent variation may influence speech characteristics. To address this, our ASR models will be trained to recognize common code-switching patterns in Singaporean English, particularly with Mandarin, Malay, and Tamil terms. During preprocessing, transcripts will be flagged for instances of code-switching, which will be analyzed both as potential confounders and as linguistically meaningful phenomena that may correlate with cognitive status.

Additionally, our machine learning approach will incorporate language background variables (primary language, education level, and self-rated proficiency) as features in model development, potentially allowing the algorithm to adjust predictions based on linguistic profile.

To address varying English proficiency among participants, we will implement several methodological controls. First, language background data will be integrated as covariates in all statistical analyses, allowing us to partial out variance attributable to language proficiency rather than cognitive status. Second, we will create individualized baseline profiles for each participant, enabling within-subject comparisons over time that are less affected by between-subject differences in language proficiency. This approach aligns with our primary aim of tracking cognitive change rather than making absolute assessments. Third, we will conduct stratified analyses based on education and language proficiency levels to determine whether different linguistic markers show varying sensitivity across these groups. For participants with lower English proficiency, we anticipate that acoustic features and simpler lexical measures may prove more reliable than complex syntactic or semantic measures.

To mitigate potential floor effects, we will normalize features within education and proficiency bands and utilize ratio measures that are more robust to education effects. Our machine learning approach will incorporate interaction terms between education/language proficiency and linguistic features, potentially revealing different cognitive-linguistic relationships across education levels. Stratified analyses will examine whether speech-cognition relationships differ between monolingual and multilingual participants, potentially revealing protective effects of multilingualism against cognitive decline, as suggested in previous research. This multi-faceted approach ensures our tools remain culturally appropriate while maintaining scientific rigor and clinical utility across Singapore’s diverse population.

### Study procedures

#### Cognitive assessment

The MoCA will be administered at each study visit. The MoCA is a widely used 30-point screening tool assessing multiple cognitive domains including memory, language, executive function, and orientation (Nasreddine et al., 2005). It has been validated in Singaporean stroke and elderly populations and is sensitive to mild PSCI (Dong et al., 2010; Khaw et al., 2021).

Montreal Cognitive Assessment scores range from 0 to 30, with greater than or equal to 26 considered normal (Carson et al., 2018). For this study, MoCA will be analyzed both as a continuous variable and dichotomized, with scores less than 26 indicating cognitive impairment.

Recognizing that education significantly influences MoCA performance, we will implement education-adjusted analyses to ensure equitable cognitive impairment classification. First, in addition to the standard cutoff of <26, we will apply education-adjusted cutoffs based on Singapore-specific normative data (Dong et al., 2010), with suggested thresholds of <25 for participants with 10–12 years of education and <22 for those with <10 years of education.

The importance of considering different cutoff values has been highlighted in recent research on stroke populations. Mirheidari et al. (2024) explored various MoCA cutoffs for detecting cognitive impairment in stroke survivors and found that while a cutoff of 26 provided balanced sensitivity and specificity, different thresholds might be optimal for specific clinical purposes. Their work suggests that education-adjusted thresholds may provide more accurate classification across diverse educational backgrounds.

In our approach, Firstly, we will conduct parallel analyses using both the standard and education-adjusted cutoffs to determine whether different classification schemes yield similar patterns of speech-cognition relationships. This approach will help identify whether certain speech markers are more robust to education effects than others. Secondly, rather than relying solely on dichotomized outcomes, we will emphasize analyses of continuous MoCA scores using regression models that explicitly include education as a covariate. This approach preserves statistical power and acknowledges the continuous nature of cognitive function. Finally, our machine learning models will incorporate education as a feature during training, potentially allowing algorithms to learn different speech-cognition relationships across education levels. Stratified performance metrics will be reported to assess whether our models achieve comparable accuracy across education bands, ensuring equitable clinical applicability.

#### Speech tasks

**Figure 1:**
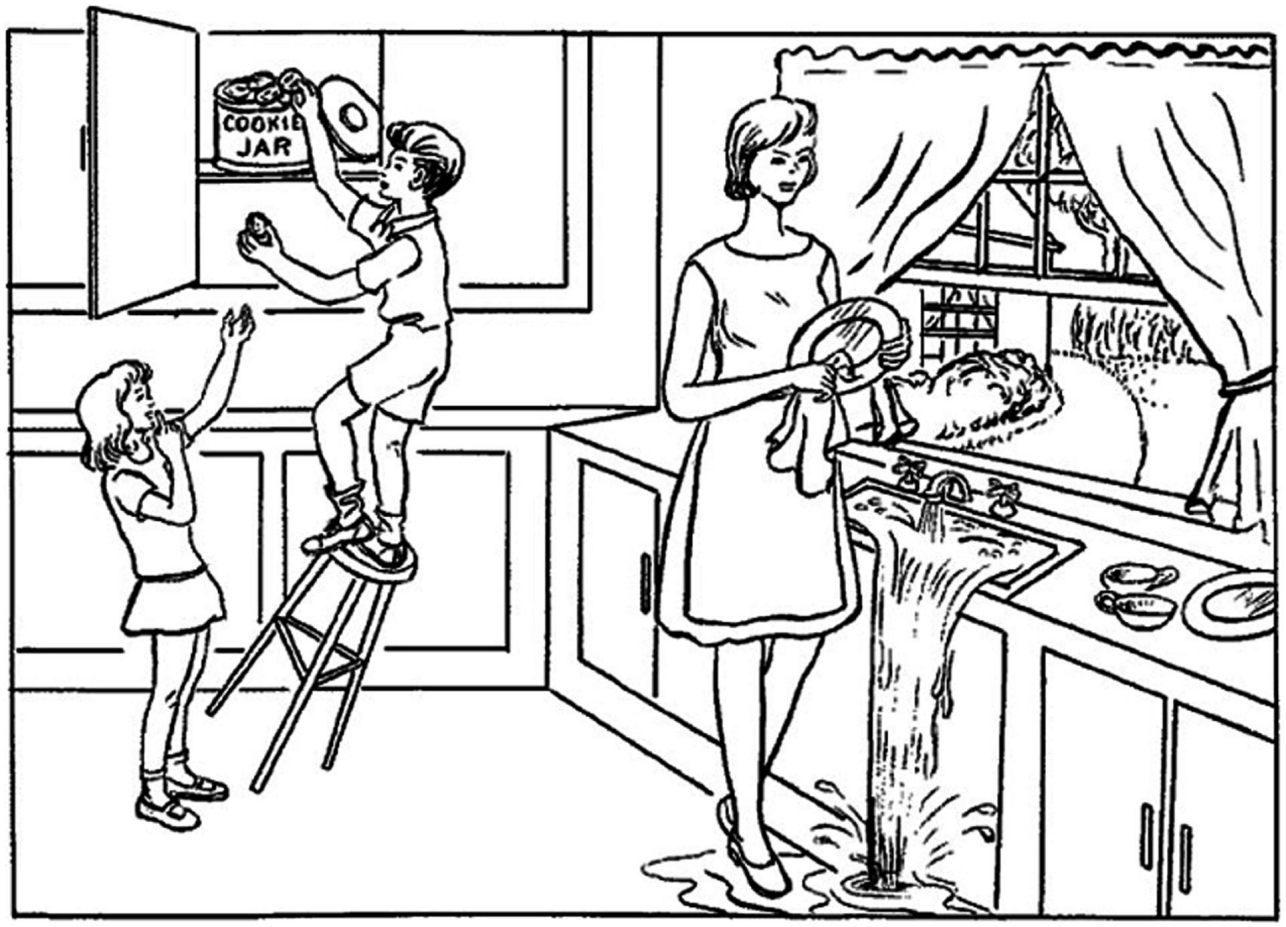
The cookie theft picture (Goodglass and Kaplan, 1983).

Speech samples will be collected using a standardized protocol comprising two tasks:

1. Picture description: Participants will be shown the Cookie Theft Picture from the Boston Diagnostic Aphasia Examination (BDAE) (Goodglass et al., 2001) on a tablet screen. This standardized assessment tool depicts a domestic kitchen scene with multiple events occurring simultaneously: two children attempting to steal cookies from a jar while precariously balanced on a stool, a woman washing dishes seemingly unaware of the overflowing sink, and various environmental details that require attention to both focal and background elements (Figure 1) (Giles and Patterson, 1996). The picture has been extensively validated for evaluating cognitive-linguistic abilities across various neurological conditions (Mueller et al., 2018), as it engages multiple cognitive domains including attention, executive function, and visuospatial processing. Studies have demonstrated its particular sensitivity to subtle changes in discourse production and semantic content in both stroke and dementia populations (Stark et al., 2021). Participants will be given standardized instructions: “Tell me everything you see happening in this picture. Try to give me as many details as you can.” While participants will be allowed up to 5 min to complete their description, most typically finish within 1–2 min (Nicholas and Brookshire, 1993). All descriptions will be audio-recorded for subsequent analysis using the specified speech processing pipeline. The Cookie Theft picture used in this study (Figure 1) is the original version from the Boston Diagnostic Aphasia Examination (BDAE) (Goodglass and Kaplan, 1983), used with appropriate permissions. While there is an updated version of this assessment tool (Berube et al., 2019), our study specifically uses the classic BDAE version due to its extensive validation across diverse neurological populations and the wealth of normative data available for comparison. This methodological choice facilitates cross-study comparisons and integration of our findings with the broader literature on cognitive-linguistic assessment. The specific version selection is important as the visual details and complexity of the scene directly influence participant responses and subsequent linguistic analysis.
2. Semi-structured conversation: Trained researchers will engage participants in a natural conversation guided by standardized open-ended questions designed to elicit spontaneous speech about personally relevant topics. The conversation will explore four key domains: the participant’s current living arrangements and family dynamics; their engagement in hobbies, interests, and social activities both before and after their stroke; significant life experiences and personal achievements; and their perspectives on aging and health. This autobiographical approach allows for natural discourse while maintaining consistency across participants through structured prompts (De Silva et al., 2025). The questions are designed to encourage extended responses and personal narrative, providing rich samples of connected speech that complement the more constrained picture description task. Researchers will be trained to use active listening techniques and minimal verbal encouragers to maximize participant speech production while minimizing their own verbal input. The semi-structured conversation will include open-ended questions designed to elicit extended discourse about personally relevant topics such as daily routines, hobbies, significant life events, and perspectives on health. Interviewers will be trained to use standardized follow-up techniques that encourage elaboration while maintaining consistency across participants.

Each task aims to elicit at least 3 min of participant speech, for a total speech sample of 6–10 min per visit. Speech recording will be standardized using calibrated Sennheiser PC8 USB headset microphones in designated quiet rooms at both sites. Recordings will be made with consistent microphone positioning and pre-session calibration. Regular acoustic environment monitoring and equipment maintenance will ensure data quality.

### Data analysis

#### Automatic speech recognition

Speech recordings will be automatically transcribed using DeepSpeech, an open-source ASR engine based on a deep neural network architecture (Hannun et al., 2014). Acoustic models will be pretrained on large, diverse Singaporean English speech corpora, including the National Speech Corpus (NSC), comprising over 1,000 h of prompted and conversational Singaporean English from multiple ethnic groups (Koh et al., 2019).

Transfer learning will be used to fine-tune the models on a subset of the study recordings, which will be manually transcribed at the word level by a team of research assistants fluent in English. Inter-transcriber reliability will be assessed using word error rate (WER) and disagreements will be resolved by consensus.

The fine-tuned models will then be applied to the remaining study recordings to generate time-aligned transcripts. Transcription accuracy will be evaluated by computing WER on a held-out validation set of manually transcribed recordings. If WER exceeds 15%, the models will be iteratively refined using data augmentation techniques such as noise and reverberation addition, speed and pitch perturbation, and accent adaptation (Ahlawat et al., 2025).

#### Linguistic feature extraction

Linguistic features will be computed from the anonymized, time-aligned transcripts using a suite of NLP tools and custom Python scripts. The following open-source libraries will be used:

- spaCy (Honnibal, 2017) for tokenization, part-of-speech tagging, dependency parsing, named entity recognition, and semantic similarity
- Natural Language Toolkit (NLTK) (Bird et al., 2009) for additional lexical diversity, readability, and sentiment analysis measures
- Gensim (Řehůřek and Sojka, 2011) for topic modeling and word embedding
- Stanford CoreNLP (Manning et al., 2014) for coreference resolution and utterance boundary detection

A comprehensive set of features will be extracted, spanning multiple levels of linguistic representation:

- Lexico-semantic:

o Word frequency and familiarity norms based on the SUBTLEX-SG database (Brysbaert et al., 2019)
o Age of acquisition and concreteness ratings based on Kuperman, Stadthagen-Gonzalez, and Brysbaert’s merged norms (Kuperman et al., 2012)
o Psycholinguistic measures such as type-token ratio, pronoun ratio, noun-verb ratio, and idea density (Lu, 2010)
o Semantic coherence metrics based on latent semantic analysis and word2vec embedding (Foltz et al., 1999)

- Morphosyntactic:

o Frequencies and ratios of major part-of-speech categories
o Measures of syntactic complexity, including mean length of utterance, clauses per utterance, parse tree height, and Yngve depth (Yngve, 1960)
o Proportions of various phrasal and clausal constructions based on parse tree patterns
o Grammatical error types and rates based on parse tree and language model anomaly detection (Foster, 2007)

- Discourse and pragmatic:

o Coherence metrics based on Centering Theory and entity grid models (Barzilay and Lapata, 2008)
o Proportion of various speech acts, including assertives, directives, commissives, and expressives (Searle, 1976)
o Dysfluency and repair rates, including filled pauses, repetitions, and retractions (Shriberg, 2001)
o Turn-taking dynamics, including mean turn length, turn switches, and overlaps (Levitan and Hirschberg, 2011)

Acoustic features will be extracted using OpenSMILE (Eyben et al., 2010), an open-source toolkit for speech signal processing. Low-level descriptors such as pitch, intensity, formants, and spectral parameters will be computed on a frame-by-frame basis and aggregated to derive global measures of prosody, voice quality, and rhythm. Specific features will include:

- Fundamental frequency (F0) statistics (mean, median, range, standard deviation)
- Jitter and shimmer (cycle-to-cycle variations in F0 and amplitude)
- Harmonic-to-noise ratio (degree of acoustic periodicity)
- Formant frequencies and bandwidths (F1, F2, F3)
- Mel-frequency cepstral coefficients (spectral envelope shape)
- Intensity and energy contours
- Speaking rate, articulation rate, and pause duration
- Stress and syllable timing patterns

All features will be standardized using *z*-scores to facilitate cross-subject comparisons. Collinear and low-variance features will be identified using correlation matrices and variance inflation factors, and removed to prevent overfitting. The final feature set will be determined based on a combination of theoretical relevance, distributional properties, and predictive power.

**Figure 2:**
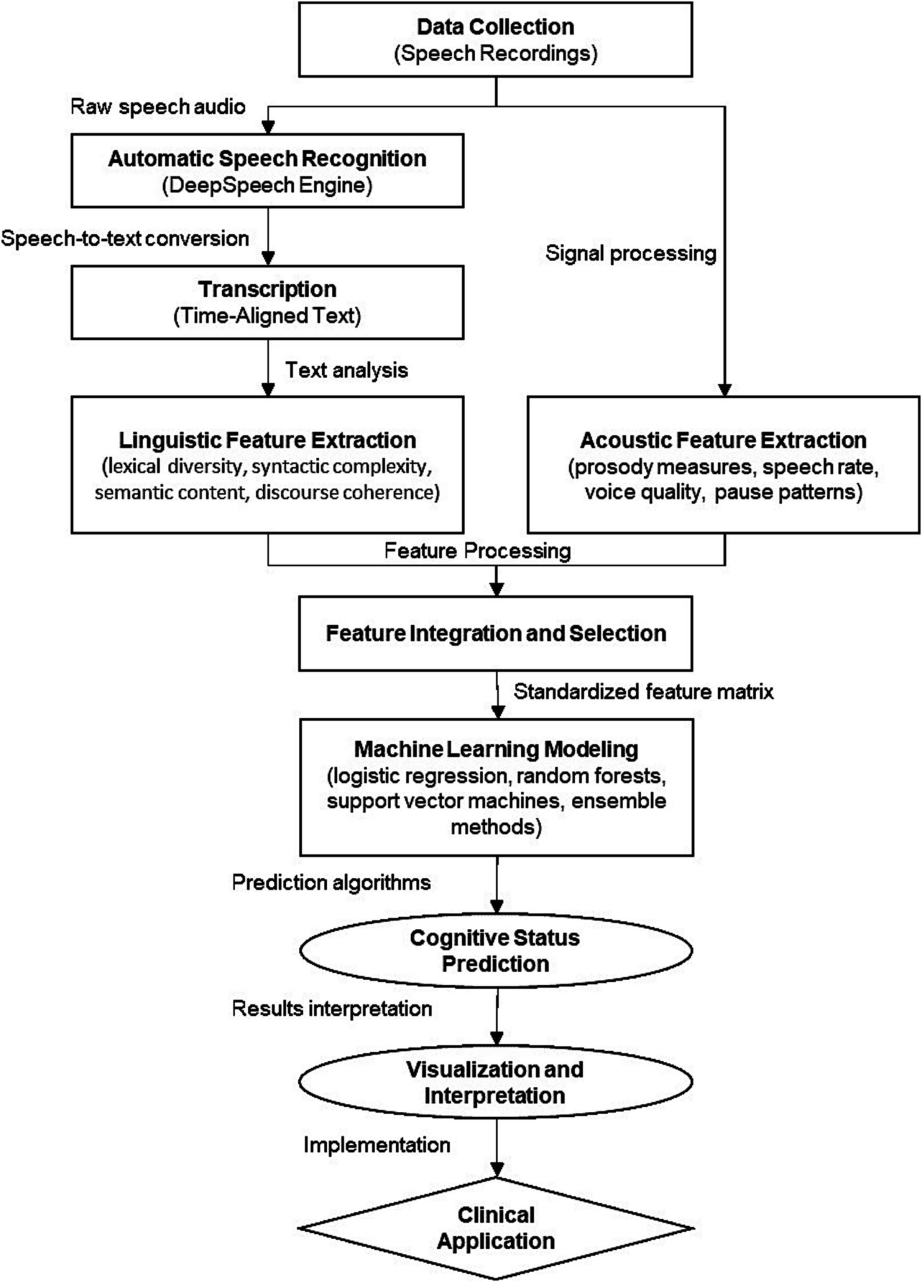
Speech analysis pipeline for PSCI detection.

Figure 2 illustrates the complete computational workflow of our speech analysis pipeline, from data collection through feature extraction to predictive modeling. This visual roadmap demonstrates the modular nature of our approach, facilitating adaptation to other clinical populations or linguistic contexts by modifying specific components while maintaining the overall analytical framework.

### Statistical analysis

Pearson correlations will be used to estimate bivariate associations between speech features and MoCA scores at each timepoint, as well as change scores across timepoints. Partial correlations adjusting for age, education, gender, and stroke severity (National Institutes of Health Stroke Scale score) will also be computed. Correlation magnitudes will be compared using Fisher’s r-to-z transformation. Statistical significance will be set at *p* < 0.05 after Benjamini-Hochberg correction for multiple comparisons.

Machine learning models will be developed to predict MoCA-defined cognitive impairment (binary classification) and MoCA scores (regression). For binary classification, logistic regression, decision trees, random forests, and support vector machines with various kernel functions will be compared. For regression, linear and regularized linear models (ridge, lasso, elastic net), decision trees, random forests, support vector regression, and Gaussian process regression will be evaluated.

Given the modest sample size, model validation will employ leave-one-out cross-validation with bootstrap resampling (1,000 iterations) for confidence interval estimation. Hyperparameter tuning will be performed within each fold using Bayesian optimization to maximize balanced accuracy. A maximum of 5 predictors will be used per model to maintain an appropriate case-to-predictor ratio. Performance metrics will be aggregated across all folds, with external validation performed using public datasets where available to assess generalizability.

The selection of the five key predictors will follow a principled, multi-stage approach combining theoretical knowledge with data-driven methods. Initially, we will categorize potential predictors into conceptual domains (lexical-semantic, syntactic, acoustic-prosodic, discourse-level, and pragmatic) based on prior literature in stroke and dementia research. Within each domain, we will identify features showing the strongest bivariate correlations with MoCA scores (|*r*| > 0.3) while demonstrating acceptable reliability (test–retest *r* > 0.7 in a subset of recordings).

Our approach is informed by recent findings from Mirheidari et al. (2024), who demonstrated that combining different feature types—particularly acoustic features capturing emotion (eGeMAPS) and linguistic features capturing contextual information (BERT)—yielded superior performance in predicting cognitive status in stroke survivors compared to using either feature set alone. This supports our strategy of selecting features across multiple linguistic and acoustic domains.

To ensure model parsimony and interpretability, feature reduction will employ a multi-stage approach. Firstly, we will eliminate features with near-zero variance or high multicollinearity (*r* > 0.85). Secondly, we will employ recursive feature elimination with cross-validation (RFECV) to identify the optimal feature subset across domains, using elastic net regularization to handle multicollinearity. This process will be constrained to select at least one feature from each domain to ensure comprehensive representation of language dimensions. Finally, we will implement principal component analysis (PCA) to identify latent dimensions underlying our feature set, retaining components explaining at least 80% of variance. For clinical interpretability, we will rotate these components and map them to functional linguistic domains (lexical access, syntactic complexity, coherence, etc.).

To enhance interpretability and clinical utility, we will prioritize features that: (1) show consistent relationships with MoCA across education and language proficiency levels; (2) demonstrate longitudinal sensitivity to cognitive change in preliminary analyses; and (3) have established neurobiological rationales linking them to cognitive processes affected by stroke.

The final predictor set will be validated through bootstrap resampling to assess stability and generalizability. If different predictor sets emerge as optimal for different subgroups (e.g., based on stroke location or education), we will develop parallel models and report comparative performance metrics to inform personalized assessment approaches.

To evaluate the performance of the machine learning models, we will use a comprehensive set of metrics. For the binary classification models predicting MoCA-defined cognitive impairment, we will calculate the area under the receiver operating characteristic curve (AUC-ROC), accuracy, sensitivity, specificity, positive predictive value, negative predictive value, and F1 score. These metrics will provide a holistic assessment of the models’ ability to discriminate between impaired and non-impaired individuals.

For the regression models predicting continuous MoCA scores, we will compute the mean absolute error (MAE), root mean square error (RMSE), coefficient of determination (R2), and Pearson correlation between the predicted and actual scores. These metrics will quantify the models’ predictive accuracy and their ability to capture the variance in cognitive performance.

95% confidence intervals for each metric will be computed using bootstrap resampling with 1,000 iterations. Pairwise comparisons between models will be conducted using dependent t-tests or Wilcoxon signed-rank tests as appropriate. Comparisons with baseline models using demographic variables alone will also be performed to assess the incremental predictive value of speech features.

The best-performing model in each category will be selected based on a holistic evaluation of performance metrics, parsimony, and interpretability. Feature importance scores will be computed using permutation importance or SHAP (SHapley Additive exPlanations) values to identify the most predictive speech markers (Breiman, 2001).

To characterize trajectories of MoCA scores and speech features over time, linear mixed-effects models will be fit using the lme4 package in R. Fixed effects will include time (in months from stroke onset) and relevant covariates such as age, gender, education, stroke type and severity (Oxfordshire Community Stroke Project classification and National Institutes of Health Stroke Scale score), lesion location, and treatment status. Random intercepts and slopes will be included to account for within-subject correlations and heterogeneity in baseline levels and rates of change. Missing data, which may occur due to participant attrition, incomplete assessments, or technical failures, will be addressed using multiple imputation by chained equations (MICE) for variables with less than 20% missingness. This approach preserves statistical power while accounting for uncertainty in imputed values. For participants with greater than 20% missing data or those lost to follow-up, sensitivity analyses will compare complete-case results with those including imputed values. Linear mixed-effects models will incorporate all available timepoints for each participant, naturally accommodating missing data under the missing-at-random assumption. Pattern-mixture models will be explored if missingness appears to be informative of cognitive status. Model selection will be performed u sing likelihood ratio tests and Akaike information criteria. Parametric bootstrapping will be used to compute 95% confidence intervals for fixed effect estimates.

The fitted models will provide estimates of the average MoCA and speech feature trajectories in the sample, as well as individual deviations from the mean trends. They will also quantify the effects of potential modifiers on these trajectories. Exploratory analyses will examine cross-lagged relationships between speech and MoCA changes to infer leadlag effects and elucidate the temporal dynamics of speech-cognition coupling.

### Acceptability and usability evaluation

At study end, all participants will be invited to participate in a semi-structured interview to evaluate their experience with the assessment protocol. The interview guide will explore four primary domains of interest: participants’ overall experience with both the MoCA and speech tasks, including their perceptions of task difficulty, comfort level during administration, and the relevance of these assessments to their daily functioning; their suggestions for optimizing task content, instructions, and administration procedures to enhance acceptability; their attitudes regarding the use of speech analysis for cognitive screening, particularly their views on its benefits, potential risks, and perceived trustworthiness compared to traditional assessment methods; and their preferences for how speech-based screening might be integrated into clinical care, including optimal frequency of administration, preferred settings, and desired formats for receiving feedback. All interviews will be audio-recorded and transcribed verbatim to ensure accurate data capture. Following qualitative research best practices (DeJonckheere and Vaughn, 2019), the interview guide will evolve through iterative refinement while maintaining consistency across interviews.

Qualitative analysis of interview transcripts will follow an inductive thematic approach based on Braun and Clarke’s six-phase framework (Braun and Clarke, 2006). Initial semantic codes will be generated through line-by-line reading, then collated into themes that capture patterns of meaning relevant to the research questions. Themes will be iteratively refined to ensure internal homogeneity and external heterogeneity. The final thematic structure will be validated through peer debriefing, member checks, and triangulation with quantitative findings. Reporting will follow the Consolidated Criteria for Reporting Qualitative Research (COREQ) (Tong et al., 2007).

NVivo software will be used to manage the coding process and maintain a detailed audit trail (Bazeley and Jackson, 2015). Rigor will be enhanced through a combination of investigator and data triangulation, thick description, reflexive journaling, and negative case analysis.

We aim to recruit a sample of 30 participants, which is feasible within the study timeframe and budget while allowing for attrition. This sample size was determined based on the exploratory aims of characterizing speech-MoCA correlations and modeling cognitive trajectories, aligning with previous proof-of-concept studies of speech biomarkers in neurocognitive disorders (Asgari et al., 2017; Alhanai et al., 2017; Themistocleous et al., 2020). While not powered for definitive diagnostic validation, this sample size will provide valuable preliminary data to inform future larger-scale research. For qualitative analyses, 30 participants is expected to achieve thematic saturation based on established guidelines (Guest et al., 2006) and our prior experience with similar patient populations.

### Ethics and dissemination

This study has been approved by the National Healthcare Group Domain Specific Review Board (NHG DSRB Ref: 2024–4,101). All participants will provide written informed consent, with the option to withdraw at any point. No monetary incentives will be provided.

Speech recordings and transcripts will be stored on secure servers with multi-factor authentication and encryption. Identifiable information will be stored separately from research data in a password-protected database accessible only to authorized study personnel. Only de-identified, aggregated data will be reported in publications.

The study team has completed training in responsible conduct of research, data privacy and security, and human subjects protection. The study will be monitored by an independent Data and Safety Monitoring Board.

Results will be disseminated through peer-reviewed publications, conference presentations, and public data sharing within 1 year of study completion. Lay summaries will be shared with participants, clinicians, and stroke support organizations.

## Discussion

This protocol describes a longitudinal pilot study to identify speech markers of post-stroke cognitive impairment (PSCI) and evaluate their prognostic utility. By leveraging state-of-the-art ASR and NLP methods, we aim to develop a sensitive, language-agnostic tool for detecting and monitoring PSCI.

Our study builds upon a growing body of research using speech analysis to detect cognitive impairment in various neurological conditions. In Alzheimer’s disease (AD), numerous studies have identified linguistic markers of cognitive decline, including reduced semantic content, syntactic complexity, and discourse coherence (Fraser et al., 2016; Mueller et al., 2018; Eyigoz et al., 2020). These features have shown promising diagnostic and predictive validity, with some models achieving over 90% accuracy in distinguishing AD from healthy controls (Orimaye et al., 2014; Noorian et al., 2017).

More recently, researchers have begun to explore speech-based cognitive assessment in stroke and vascular dementia. Corbett et al. (2009) found that measures of lexical diversity and content word frequency could discriminate between patients with PSCI and healthy controls. Tomoeda et al. (1996) demonstrated that semantic content during picture description was associated with overall cognitive function in stroke survivors. Pou-Prom and Rudzicz (2018) used word embedding to identify stroke patients with or without cognitive impairment based on spontaneous speech transcripts.

However, most previous studies have been cross-sectional and focused on group-level differences rather than individual prediction. They have also been limited by small, heterogeneous samples, manual transcription methods, and a narrow range of language features. In contrast, our study employs a longitudinal design, automated speech processing, and a comprehensive feature set to characterize both between-person and within-person variation in speech and cognition over time. Our focus on the subacute-to-chronic stages of stroke recovery addresses a critical gap, as most research to date has examined the acute phase.

Methodologically, our approach draws upon best practices for ASR development, such as transfer learning, data augmentation, and multi-level evaluation (Xiong et al., 2018). The use of Transformer-based acoustic models pretrained on large, multilingual corpora is expected to yield high transcription accuracy for Singaporean English. Extensive feature engineering guided by linguistic theory captures the multidimensional nature of language impairment in PSCI.

Our analytical framework also extends previous work by integrating modern machine learning techniques with traditional longitudinal modeling. The use of elastic net regularization, nested cross-validation, and bootstrap CIs helps guard against overfitting and enhances the reliability of predictive models (Zou and Hastie, 2005; Cawley and Talbot, 2010). Mixed effects models offer a flexible way to estimate both population-averaged and subject-specific cognitive trajectories while accounting for missing data.

Qualitatively, our study is among the first to explore the acceptability and usability of speech-based cognitive assessment from the patient perspective. While a few studies have examined user experiences with digital tools for stroke rehabilitation (Tatla et al., 2015), none have focused specifically on speech analysis or cognitive screening. Insights from participant interviews will inform the design of future speech-based interventions to maximize engagement and adherence.

A key strength of this study is the interdisciplinary team, which combines expertise in stroke neurology, neuropsychology, linguistics, and AI. This allows us to approach the problem of PSCI detection from multiple angles and develop a solution that is both technically robust and clinically meaningful. The study also benefits from Singapore’s diverse, multilingual population and advanced technological infrastructure.

However, some limitations should be acknowledged. First, the modest sample size may limit statistical power, especially for detecting interaction effects or subgroup differences. We have tried to mitigate this through a parsimonious modeling approach and the use of bias-corrected CIs. Second, as a single-center study, findings may have limited generalizability to other settings. Multi-site validation using a common protocol would help establish external validity. Third, while the MoCA is a well-validated screening tool, it is not a substitute for comprehensive neuropsychological testing. Incorporation of additional domain-specific tests could provide a more nuanced picture of cognitive deficits.

Another potential limitation is the use of semi-controlled speech tasks, which may not fully capture the richness and complexity of spontaneous discourse. However, these tasks are necessary to ensure comparability across participants and time points. They also simulate the types of questions commonly asked during clinical encounters. Future studies could explore the use of free conversation or narrative storytelling to elicit more naturalistic speech samples.

Finally, as an observational study, we cannot directly infer causal relationships between speech features and cognitive outcomes. The proposed analyses can only establish associations and generate hypotheses for future testing. Experimental designs that manipulate speech parameters or compare different assessment modalities would provide stronger evidence of causality.

Despite these caveats, our study has important implications for research and practice. Methodologically, it demonstrates the feasibility and utility of combining NLP and ML techniques to analyze speech data at scale. The proposed feature engineering and modeling pipelines could be readily adapted to other languages, accents, and neurological conditions. Clinically, our findings could inform the development of speech-based screening tools for early detection and monitoring of PSCI. Such tools could be integrated into telemedicine platforms or mobile apps, enabling remote cognitive assessment between clinic visits. This could help optimize resource allocation, identify high-risk patients, and evaluate the effects of interventions.

In the longer term, speech biomarkers could serve as objective, language-agnostic endpoints for clinical trials of novel therapies for PSCI. They could also be combined with other digital markers such as gait, sleep, social media activity and so on to create multi-modal risk scores and care pathways. As speech interfaces become increasingly ubiquitous, there will be even greater opportunities to harness natural language interactions for health monitoring.

Future studies should aim to replicate and extend our findings in larger, more diverse cohorts. This will require close collaboration among stroke centers to harmonize data collection and processing methods. More work is also needed to establish the minimal clinically important differences and predictive values of speech biomarkers, as well as their incremental utility over traditional cognitive tests. User-centered design principles should guide the translation of research findings into practical tools that are easy to use and interpret.

This study represents an important step toward a vision of personalized, precision medicine for stroke recovery. By harnessing the power of artificial intelligence and real-world language data, we can develop more sensitive, efficient, and equitable approaches to detecting and treating PSCI. Our hope is that this work will not only advance scientific understanding of post-stroke cognition, but also make a meaningful difference in the lives of stroke survivors.

Specifically, this approach could enable earlier detection of subtle cognitive changes before they manifest as functional impairment, allowing for timely initiation of cognitive rehabilitation therapies and secondary stroke prevention measures. For patients, automated speech analysis could reduce the burden of frequent in-person clinical visits by enabling remote cognitive monitoring through telephone or video calls, particularly valuable for those with mobility limitations or living in areas with limited access to specialists. The technology could also provide more frequent assessment points without increasing clinician workload, creating a more continuous picture of cognitive trajectories rather than the widely-spaced snapshots afforded by traditional testing. Additionally, by detecting domain-specific cognitive changes with greater sensitivity than global screening tools, this approach could enable more personalized rehabilitation strategies targeting specific cognitive weaknesses.

## Glossary

AD: Alzheimer’s disease
AI: artificial intelligence
ASR: automatic speech recognition
AUC-ROC: area under the receiver operating characteristic curve
BDAE: Boston diagnostic aphasia examination
CI: confidence interval
COREQ: consolidated criteria for reporting qualitative research
F0: fundamental frequency
F1: first formant
F2: second formant
F3: third formant
MAE: mean absolute error
ML: machine learning
MMSE: mini-mental state examination
MoCA: Montreal cognitive assessment
NHG DSRB: National Healthcare Group Domain Specific Review Board
NLP: natural language processing
NLTK: natural language toolkit
NSC: national speech corpus
PSCI: post-stroke cognitive impairment
RMSE: root mean square error
SHAP: Shapley additive explanations
SUBTLEX-SG: Singapore subtitle word frequency database
WER: word error rate

## References

[ref1] Ahlawat H. Aggarwal N. Gupta D. (2025). Automatic speech recognition: a survey of deep learning techniques and approaches. Int. J. Cognit. Comput. Engin. 6, 201–237. doi: 10.1016/j.ijcce.2024.12.007

[ref2] Akkad H. Hope T. M. H. Howland C. Ondobaka S. Pappa K. Nardo D. . (2023). Mapping spoken language and cognitive deficits in post-stroke aphasia. NeuroImage 39:103452. doi: 10.1016/j.nicl.2023.103452, PMID: 37321143 PMC10275719

[ref3] Alhanai T. Au R. Glass J. (2017). Spoken language biomarkers for detecting cognitive impairment. In 2017 IEEE automatic speech recognition and understanding workshop (ASRU). 409–416.

[ref4] Alhashimi A. Kamarova M. Baig S. S. Nair K. P. S. Wang T. Redgrave J. . (2024). Remote ischaemic conditioning for neurological disorders—a systematic review and narrative synthesis. Syst. Rev. 13:308. doi: 10.1186/s13643-024-02725-8, PMID: 39702489 PMC11657452

[ref5] Asgari M. Kaye J. Dodge H. (2017). Predicting mild cognitive impairment from spontaneous spoken utterances. Alzheimers Dement. 3, 219–228. doi: 10.1016/j.trci.2017.01.006, PMID: 29067328 PMC5651423

[ref6] Barzilay R. Lapata M. (2008). Modeling local coherence: an entity-based approach. Comput. Linguist. 34, 1–34. doi: 10.1162/coli.2008.34.1.1

[ref7] Bazeley B. C. P. Jackson K. (2015). Qualitative data analysis with NVivo. Qual. Res. Psychol. 12, 492–494.

[ref8] Beltrami D. Gagliardi G. Rossini Favretti R. Ghidoni E. Tamburini F. Calzà L. (2018). Speech analysis by natural language processing techniques: a possible tool for very early detection of cognitive decline? Front. Aging Neurosci. 10:369. doi: 10.3389/fnagi.2018.00369, PMID: 30483116 PMC6243042

[ref9] Berube S. Nonnemacher J. Demsky C. Glenn S. Saxena S. Wright A. . (2019). Stealing cookies in the twenty-first century: measures of spoken narrative in healthy versus speakers with aphasia. Am. J. Speech Lang. Pathol. 28, 321–329. doi: 10.1044/2018_AJSLP-17-0131, PMID: 30242341 PMC6437702

[ref10] Bird S. Klein E. Loper E. (2009). Natural language processing with Python: Analyzing text with the natural language toolkit. Sebastopol, CA, United States: O'Reilly Media, Inc.

[ref11] Braun V. Clarke V. (2006). Using thematic analysis in psychology. Qual. Res. Psychol. 3, 77–101. doi: 10.1191/1478088706qp063oa

[ref12] Breiman L. (2001). Random forests. Mach. Learn. 45, 5–32. doi: 10.1023/A:1010933404324

[ref13] Brysbaert M. Mandera P. McCormick S. F. Keuleers E. (2019). Word prevalence norms for 62,000 English lemmas. Behav. Res. Methods 51, 467–479. doi: 10.3758/s13428-018-1077-9, PMID: 29967979

[ref14] Carson N. Leach L. Murphy K. J. (2018). A re-examination of Montreal cognitive assessment (MoCA) cutoff scores. Int. J. Geriatr. Psychiatry 33, 379–388. doi: 10.1002/gps.4756, PMID: 28731508

[ref15] Cawley G. Talbot N. (2010). On over-fitting in model selection and subsequent selection Bias in performance evaluation. J. Mach. Learn. Res. 11, 2079–2107.

[ref16] Corbett F. Jefferies E. Ehsan S. Lambon Ralph M. A. (2009). Different impairments of semantic cognition in semantic dementia and semantic aphasia: evidence from the non-verbal domain. Brain 132, 2593–2608. doi: 10.1093/brain/awp146, PMID: 19506072 PMC2766180

[ref17] de la Fuente G. S. Ritchie C. W. Luz S. (2020). Artificial intelligence, speech, and language processing approaches to monitoring Alzheimer’s disease: a systematic review. J. Alzheimers Dis. 78, 1547–1574. doi: 10.3233/JAD-200888, PMID: 33185605 PMC7836050

[ref18] De Silva U. Madanian S. Olsen S. Templeton J. M. Poellabauer C. Schneider S. L. . (2025). Clinical decision support using speech signal analysis: systematic scoping review of neurological disorders. J. Med. Internet Res. 27:e63004. doi: 10.2196/63004, PMID: 39804693 PMC11773292

[ref19] DeJonckheere M. Vaughn L. M. (2019). Semistructured interviewing in primary care research: a balance of relationship and rigour. Fam. Med. Commun. Health 7:e000057. doi: 10.1136/fmch-2018-000057, PMID: 32148704 PMC6910737

[ref20] Dong Y. Sharma V. K. Chan B. P. Venketasubramanian N. Teoh H. L. Seet R. C. . (2010). The Montreal cognitive assessment (MoCA) is superior to the Mini-mental state examination (MMSE) for the detection of vascular cognitive impairment after acute stroke. J. Neurol. Sci. 299, 15–18. doi: 10.1016/j.jns.2010.08.051, PMID: 20889166

[ref21] Eyben F. Wöllmer M. Schuller B. (2010). openSMILE: the Munich versatile and fast open-source audio feature extractor. In Proceedings of the 18th ACM international conference on multimedia. 1459–1462.

[ref22] Eyigoz E. Mathur S. Santamaria M. Cecchi G. Naylor M. (2020). Linguistic markers predict onset of Alzheimer's disease. EClinicalMedicine. 28:100583. doi: 10.1016/j.eclinm.2020.100583, PMID: 33294808 PMC7700896

[ref23] Foltz P Laham D Landauer T. Automated essay scoring: applications to educational technology. World Conference on Educational Multimedia, Hypermedia and Telecommunications. eds. Colli, B and Oliver, R. Seattle, Washington, USA, Charlottesville, VA: Association for the Advancement of Computing in Education. (1999) 1, 19–24.

[ref24] Foster J. (2007). Treebanks gone bad. IJDAR 10, 129–145. doi: 10.1007/s10032-007-0059-8, PMID: 40260408

[ref25] Fraser K. C. Meltzer J. A. Rudzicz F. (2016). Linguistic features identify Alzheimer's disease in narrative speech. J. Alzheimers Dis. 49, 407–422. doi: 10.3233/JAD-150520, PMID: 26484921

[ref26] Giles E. Patterson K. (1996). Performance on the Boston cookie theft picture description task in patients with early dementia of the Alzheimer's type: missing information. Aphasiology 10, 395–408. doi: 10.1080/02687039608248419

[ref27] Goodglass H. Kaplan E. (1983). Boston diagnostic aphasia examination booklet. Philadelphia, Pennsylvania: Lea & Febiger.

[ref28] Goodglass H. Kaplan E. Weintraub S. (2001). BDAE: The Boston diagnostic aphasia examination. Philadelphia, PA: Lippincott Williams & Wilkins.

[ref29] Guest G. Bunce A. Johnson L. (2006). How many interviews are enough? Field Methods 18, 59–82. doi: 10.1177/1525822X05279903

[ref30] Hannun A Case C Casper J Catanzaro B Diamos G Elsen E . DeepSpeech: Scaling up end-to-end speech recognition. arXiv preprint, arXiv:1412.5567. (2014).

[ref31] Honnibal M. spaCy 2: Natural language understanding with bloom embeddings, convolutional neural networks and incremental parsing. (2017)

[ref32] Kalaria R. N. Akinyemi R. Ihara M. (2016). Stroke injury, cognitive impairment and vascular dementia. Biochim. Biophys. Acta (BBA) - Mol. Basis Dis. 1862, 915–925. doi: 10.1016/j.bbadis.2016.01.015, PMID: 26806700 PMC4827373

[ref33] Khaw J. Subramaniam P. Abd Aziz N. A. Ali Raymond A. Wan Zaidi W. A. Ghazali S. E. (2021). Current update on the clinical utility of MMSE and MoCA for stroke patients in Asia: a systematic review. Int. J. Environ. Res. Public Health 18:8962. doi: 10.3390/ijerph18178962, PMID: 34501552 PMC8431226

[ref34] Koh J. Mislan A. Khoo K. Ang B. Ang W. Ng C. . (2019). Building the Singapore English National Speech Corpus, 321–325. doi: 10.21437/Interspeech.2019-1525

[ref35] König A. Satt A. Sorin A. Hoory R. Toledo-Ronen O. Derreumaux A. . (2015). Automatic speech analysis for the assessment of patients with predementia and Alzheimer's disease. Alzheimers Dement. 1, 112–124. doi: 10.1016/j.dadm.2014.11.012, PMID: 27239498 PMC4876915

[ref36] Kuperman V. Stadthagen-Gonzalez H. Brysbaert M. (2012). Age-of-acquisition ratings for 30,000 English words. Behav. Res. Methods 44, 978–990. doi: 10.3758/s13428-012-0210-4, PMID: 22581493

[ref37] Lee M. Yeo N.-Y. Ahn H.-J. Lim J.-S. Kim Y. Lee S.-H. . (2023). Prediction of post-stroke cognitive impairment after acute ischemic stroke using machine learning. Alzheimers Res. Ther. 15:147. doi: 10.1186/s13195-023-01289-4, PMID: 37653560 PMC10468853

[ref38] Levitan R. Hirschberg J. (2011). Measuring acoustic-prosodic entrainment with respect to multiple levels and dimensions. In Interspeech. 3081–3084.

[ref39] Lu X. (2010). Automatic analysis of syntactic complexity in second language writing. Int. J. Corpus Linguist. 15, 474–496. doi: 10.1075/ijcl.15.4.02lu

[ref40] Manning C Surdeanu M Bauer J Finkel J Bethard S Mcclosky D. The Stanford CoreNLP natural language processing toolkit (2014)

[ref41] Martínez-Nicolás I. Llorente T. E. Martínez-Sánchez F. Meilán J. J. G. (2021). Ten years of research on automatic voice and speech analysis of people with Alzheimer's disease and mild cognitive impairment: a systematic review article. Front. Psychol. 12:620251. doi: 10.3389/fpsyg.2021.620251, PMID: 33833713 PMC8021952

[ref42] Mirheidari B. Bell S. M. Harkness K. Blackburn D. Christensen H. (2024). Spoken language-based automatic cognitive assessment of stroke survivors. Lang. Health 2, 32–38. doi: 10.1016/j.laheal.2024.01.001

[ref43] Mueller K. D. Hermann B. Mecollari J. Turkstra L. S. (2018). Connected speech and language in mild cognitive impairment and Alzheimer's disease: a review of picture description tasks. J. Clin. Exp. Neuropsychol. 40, 917–939. doi: 10.1080/13803395.2018.1446513, PMID: 29669461 PMC6198327

[ref44] Nasreddine Z. S. Phillips N. A. Bédirian V. Charbonneau S. Whitehead V. Collin I. . (2005). The Montreal cognitive assessment, MoCA: a brief screening tool for mild cognitive impairment. J. Am. Geriatr. Soc. 53, 695–699. doi: 10.1111/j.1532-5415.2005.53221.x, PMID: 15817019

[ref45] Nicholas L. E. Brookshire R. H. (1993). A system for quantifying the informativeness and efficiency of the connected speech of adults with aphasia. J. Speech Hear. Res. 36, 338–350. doi: 10.1044/jshr.3602.338, PMID: 8487525

[ref46] Noorian Z Pou-Prom C Rudzicz F. On the importance of normative data in speech-based assessment. arXiv preprint, arXiv:1712.00069. (2017).

[ref47] Orimaye S Wong J Golden K. Learning predictive linguistic features for Alzheimer's disease and related dementias using verbal utterances. In Proceedings of the workshop on computational linguistics and clinical psychology: from linguistic signal to clinical reality. (2014).

[ref48] Pendlebury S. T. Rothwell P. M. (2009). Prevalence, incidence, and factors associated with pre-stroke and post-stroke dementia: a systematic review and meta-analysis. Lancet Neurol. 8, 1006–1018. doi: 10.1016/S1474-4422(09)70236-4, PMID: 19782001

[ref49] Pou-Prom C. Rudzicz F. (2018). Learning multiview embeddings for assessing dementia. In Proceedings of the 2018 conference on empirical methods in natural language processing. 2812–2817.

[ref50] Řehůřek R Sojka P. (2011). Gensim—statistical semantics in python. Available at: https://radimrehurek.com/gensim/

[ref51] Robin J. Harrison J. E. Kaufman L. D. Rudzicz F. Simpson W. Yancheva M. (2020). Evaluation of speech-based digital biomarkers: review and recommendations. Digit Biomark. 4, 99–108. doi: 10.1159/000510820, PMID: 33251474 PMC7670321

[ref52] Searle J. R. (1976). A classification of illocutionary acts. Lang. Soc. 5, 1–23. doi: 10.1017/S0047404500006837

[ref53] Shriberg E. (2001). To 'errrr' is human: ecology and acoustics of speech disfluencies. J. Int. Phon. Assoc. 31, 153–169. doi: 10.1017/S0025100301001128

[ref54] Simon B. Bahman M. Kirsty H. Mary S. Jonathan G. Madalina R. . (2024). COGNOSPEAK: a feasibility pilot study of automated speech analysis to aid cognitive assessment post stroke. J. Neurol. Neurosurg. Psychiatry 95:A38. doi: 10.1136/jnnp-2024-ABN.124

[ref55] Stark B. C. Dutta M. Murray L. L. Bryant L. Fromm D. MacWhinney B. . (2021). Standardizing assessment of spoken discourse in aphasia: a working group with deliverables. Am. J. Speech Lang. Pathol. 30, 491–502. doi: 10.1044/2020_AJSLP-19-00093, PMID: 32585117 PMC9128722

[ref56] Sun J. H. Tan L. Yu J. T. (2014). Post-stroke cognitive impairment: epidemiology, mechanisms and management. Ann. Transl. Med. 2:80. doi: 10.3978/j.issn.2305-5839.2014.08.05, PMID: 25333055 PMC4200648

[ref57] Tang E. Y. Amiesimaka O. Harrison S. L. Green E. Price C. Robinson L. . (2018). Longitudinal effect of stroke on cognition: a systematic review. J. Am. Heart Assoc. 7:e006443. doi: 10.1161/JAHA.117.006443, PMID: 29335318 PMC5850140

[ref58] Tatla S. K. Shirzad N. Lohse K. R. Virji-Babul N. Hoens A. M. Holsti L. . (2015). Therapists' perceptions of social media and video game technologies in upper limb rehabilitation. JMIR Serious Games 3:e2. doi: 10.2196/games.3401, PMID: 25759148 PMC4373832

[ref59] Teh W. L. Abdin E. Vaingankar J. A. Seow E. Sagayadevan V. Shafie S. . (2018). Prevalence of stroke, risk factors, disability and care needs in older adults in Singapore: results from the WiSE study. BMJ Open 8:e020285. doi: 10.1136/bmjopen-2017-020285, PMID: 29599393 PMC5875611

[ref60] Themistocleous C. Eckerström M. Kokkinakis D. (2020). Voice quality and speech fluency distinguish individuals with mild cognitive impairment from healthy controls. PLoS One 15:e0236009. doi: 10.1371/journal.pone.0236009, PMID: 32658934 PMC7357785

[ref61] Tomoeda C. K. Bayles K. A. Trosset M. W. Azuma T. McGeagh A. (1996). Cross-sectional analysis of Alzheimer disease effects on oral discourse in a picture description task. Alzheimer Dis. Assoc. Disord. 10, 204–215. doi: 10.1097/00002093-199601040-00006, PMID: 8939280

[ref62] Tong A. Sainsbury P. Craig J. (2007). Consolidated criteria for reporting qualitative research (COREQ): a 32-item checklist for interviews and focus groups. Int. J. Qual. Health Care 19, 349–357. doi: 10.1093/intqhc/mzm042 17872937

[ref63] Venketasubramanian N. Yoon B. W. Pandian J. Navarro J. C. (2017). Stroke epidemiology in south, east, and South-East Asia: a review. J. Stroke 19, 286–294. doi: 10.5853/jos.2017.00234, PMID: 29037005 PMC5647629

[ref64] Xiong W. Wu L. Alleva F. Droppo J. Huang X. Stolcke A. . (2018). The Microsoft 2017 conversational speech recognition system. 2018 IEEE international conference on acoustics, speech and signal processing (ICASSP). IEEE. 15–20.

[ref65] Yngve V. H. (1960). A model and an hypothesis for language structure. Proc. Am. Philos. Soc. 104, 444–466.

[ref66] Zhao Y. Halai A. D. Lambon Ralph M. A. (2020). Evaluating the granularity and statistical structure of lesions and behaviour in post-stroke aphasia. Brain Commun. 2:fcaa062. doi: 10.1093/braincomms/fcaa062, PMID: 32954319 PMC7472896

[ref67] Zietemann V. Georgakis M. K. Dondaine T. Müller C. Mendyk A. M. Kopczak A. . (2018). Early MoCA predicts long-term cognitive and functional outcome and mortality after stroke. Neurology 91, e1838–e1850. doi: 10.1212/WNL.0000000000006506, PMID: 30333158

[ref68] Zou H. Hastie T. (2005). Regularization and variable selection via the elastic net. J. R. Statistic. Soc. Series B 67, 301–320. doi: 10.1111/j.1467-9868.2005.00503.x

